# Elevated circulating soluble interleukin-2 receptor in patients with chronic liver diseases is associated with non-classical monocytes

**DOI:** 10.1186/1471-230X-12-38

**Published:** 2012-04-24

**Authors:** Sebastian Seidler, Henning W Zimmermann, Ralf Weiskirchen, Christian Trautwein, Frank Tacke

**Affiliations:** 1 Department of Medicine III, University Hospital, RWTH-Aachen, Germany; 2 Institute of Clinical Chemistry and Pathobiochemistry, University Hospital, RWTH-Aachen, Germany

**Keywords:** Liver cirrhosis, Liver fibrosis, Interleukin-2, CD25, Monocytes, Macrophages

## Abstract

**Background:**

The soluble interleukin-2 receptor (sIL-2R, sIL2R, sTAC, sCD25) is a reliable biomarker for disease activity in inflammatory disorders such as sarcoidosis. Based on the essential pathogenic role of inflammation for progression of liver diseases, we hypothesized that sIL-2R might be an indicator of inflammatory cell activation and disease severity in patients with chronic liver diseases (CLD).

**Methods:**

We measured sIL-2R serum levels in 71 patients with different stages and etiologies of CLD in comparison to 41 healthy controls. Serum sIL-2R concentrations were correlated with laboratory markers of liver diseases, cytokine / chemokine levels and circulating immune cell subpopulations as simultaneously assessed by FACS analysis from peripheral leukocytes.

**Results:**

CLD patients showed significantly elevated serum sIL-2R levels compared with controls. sIL-2R was significantly higher in patients with compared to patients without established liver cirrhosis and increased with the Child-Pugh stage of cirrhosis, independent of the underlying etiology. sIL-2R levels correlated inversely with parameters indicating the hepatic biosynthetic capacity, such as albumin or international normalized ratio, and positively with non-invasive markers of liver fibrosis such as hyaluronic acid or procollagen-III-peptide. Circulating immune cells might represent a major source of sIL-2R. In fact, sIL2-R levels correlated closely with circulating monocytes, especially non-classical CD14+ CD16+ monocytes, which were found to express high levels of CD25 by FACS. Pro-inflammatory cytokines, including IL-2, IFNγ or IL-6, and chemokines were also associated with sIL2-R. In addition, renal failure was an important confounder of sIL-2R levels independent of liver dysfunction and inflammation.

**Conclusions:**

sIL-2R is elevated in patients with liver diseases and cirrhosis, is associated with circulating inflammatory cells and is increased in concomitant renal failure. These data indicate that sIL-2R might be a potential marker for immune cell activation in CLD, especially for proinflammatory and profibrogenic non-classical CD14 + CD16+ monocytes.

## Background

Since its discovery in 1985 the soluble interleukin-2 receptor (sIL-2R, sTAC, sCD25) has become a clinically valuable tool for several diseases [1]. It is regarded as a disease activity marker in sarcoidosis [2,3], but increased serum levels have been also observed in other autoimmune diseases like systemic lupus erythematosus and rheumatoid arthritis [4]. In addition, sIL-2R is elevated in several neoplastic disorders, and it appears useful in estimating survival and monitoring therapy in malignancies like malignant melanoma or nasopharyngeal carcinoma [5,6].

While interleukin-2 is primarily secreted by activated T-helper lymphocytes [7,8], the interleukin-2 receptor (IL-2R, CD25) is widely expressed among many leukocytes. Although activated T lymphocytes and regulatory T cells express high levels of IL-2Rα, which is part of the IL-2receptor, on their surface [8-12], it is well known that activated B cells, monocytes, eosinophil granulocytes and natural killer cells (NK cells) also express CD25 [13-17]. The soluble form of the IL-2 receptor seems to be produced by proteolytic cleavage of IL-2Rα, and the release of sIL-2R into the circulation has been found to be proportional to its membrane bound expression [3,16,18]. This is regarded as the main reason why sIL2-R is a reliable biomarker for disease activity in inflammatory disorders, especially in sarcoidosis and other autoimmune diseases.

Due to its close association with inflammatory processes, sIL-2R could be possibly a useful marker in chronic liver diseases (CLD), given the fact that chronic inflammation is believed to be the key driver for disease progression [19,20]. There are some studies examining sIL-2R in CLD suggesting increased sIL-2R in hepatic disorders [21,22]. However, a major drawback of these studies is that they focused on selected etiologies, predominantly viral-related liver diseases or primary biliary cirrhosis [21-24]. It thus remained unclear whether sIL-2R may be universally useful to monitor inflammatory activities in progressing CLD and to which extent sIL-2R may reflect activation of distinct leukocyte subpopulations in CLD.

In the current work we measured sIL-2R levels in 71 patients with different stages and a wide spectrum of etiologies of chronic hepatic disorders in comparison to 41 healthy controls. We analyzed whether sIL-2R serum levels correlated with clinical and routine laboratory markers of liver diseases, but also with experimental cytokine and chemokine levels or circulating immune cell subpopulations as simultaneously assessed by FACS analysis from peripheral leukocytes. We hypothesized that sIL-2R might be a general indicator of inflammatory cell activation in CLD patients.

## Materials & methods

### Study participants

The local ethics committee (ethics committee of University Hospital Aachen, RWTH Aachen) approved the study protocol, and written informed consent was obtained from each participant. Patients (n = 71) with chronic liver disease (CLD) independent of the etiology were enrolled in the present study. Patient characteristics are summarized in Table 1. 48 patients showed overt signs for cirrhosis confirmed by imaging (MRI, CT scan or ultrasound) or cirrhosis related complications (e.g. esophageal varices or encephalopathy) [25]. Patients with acute liver failure, acute hepatitis, HIV infection, bacterial infection (procalcitonin above 0.5 μg/l) and systemic steroid medication were excluded. Furthermore, patients with malignant tumors and with hepatocellular carcinoma were excluded, because malignancies are known to increase sIL2-R levels [5,6]. Healthy controls (n = 41) were recruited from the local blood transfusion institute and from the staff of the Department of Medicine III of the University Hospital Aachen, Germany. They were tested negatively for HIV, HBV and HCV infections. Peripheral blood samples were collected from all study participants by venipuncture. Blood was processed for immediate FACS analysis, while sera were stored at −80°C until analysis.

**Table 1:** Patient characteristics and sIL-2R measurements

	**controls**	**all patients**	**stage of cirrhosis**			
			no cirrh.	Child A	Child B	Child C
n	41	71	23	21	13	14
sex male/ female n	20/21	37/34	11/12	8/13	8/5	10/4
age yrs	35	54	43	61	63	53
median (range)	(18–65)	(17–74)	(17–64)	(30–74)	(28–74)	(34–73)
disease etiology n	n.a.					
virus hepatitis		24	11	8	5	0
biliary/autoimmune		8	2	4	1	1
alcohol		21	3	5	3	10
other origin		18	7	4	4	3
MELD score	n.a.	n.a.	n.a.	8	11	17
median (range)				(6–17)	(7–16)	(12–28)
ALT U/L	19	41	64	34	36	29
median (range)	(5–54)	(7–568)	(15–568)	(8–188)	(7–92)	(14–261)
bilirubin mg/dL	n.a.	1.3	0.7	0.9	1.6	5.1
median (range)		(0.2-12.5)	(0.4-8.7)	(0.2-8.9)	(0.2-9.0)	(1.8-12.5)
albumin mg/dL	n.a.	38	45	40	27	27
median (range)		(12–50)	(40–50)	(30–43)	(12–38)	(22–31)
international normalized	n.a.	1.07	0.98	1.06	1.20	1.43
ratio median (range)		(0.92-1.69)	(0.93-1.12)	(0.92-1.32)	(0.99-1.39)	(1.18-1.69)
hyaluronic acid μg/l	n.a.	190	28	205	305	800
median (range)		(10–800)	(10–210)	(36–800)	(140–800)	(190–800)
procollagen-III-peptide	584	1135	973	990	1710	2500
median (range) μg/l	(157–1160)	(470–6560)	(470–1750)	(589–1930)	(639–2630)	(1010–6560)
creatinine mg/dL	0.7	0.7	0.7	0.7	0.6	0.8
median (range)	(0.6-0.9)	(0.3-2.4)	(0.4-1.0)	(0.4-1.9)	(0.4-1.8)	(0.3-2.4)
cystatin C mg/L	n.a.	0.98	0.80	1.01	1.20	1.46
median (range)		(0.40-2.81)	(0.40-1.16)	(0.69-2.81)	(0.74-2.78)	(0.80-2.41)
GFR [cyst. C] ml/min	n.a.	88	123	83	64	45
median (range)		(15–395)	(66–395)	(15–158)	(15–140)	(19–123)
total monocytes x10^6^/L	416	490	419	426	477	816
median (range)	(165–852)	(70–2208)	(70–860)	(296–1332)	(162–2208)	(294–1180)
CD14^++^CD16^-^ monocytes	386	442	384	387	425	714
x10^6^/L median (range)	(155–748)	(65–2101)	(65–807)	(250–1155)	(142–2101)	(257–1089)
CD14^+^CD16^+^ monocytes	32.4	46	40	45	67	92
x10^6^/L median (range)	(10–108)	(4.6-175)	(4.6-158)	(26–175)	(18–135)	(6.4-153)
sIL-2R kU/L	444	818	374	764	1101	1029
median (range)	(14–1193)	(39–3976)	(204–1795)	(39–3976)	(317–2494)	(370–3350)

ALT, alanine aminotransferase activity; GFR, glomerular filtration rate; MELD, model of end-stage liver disease; n.a., not applicable / not assessed; sIL-2R, soluble interleukin-2 receptor.

### Analysis of circulating leukocytes via flow cytometry

Peripheral blood mononuclear cells (PBMC) were isolated by Ficoll Density Gradient, as described before [26,27]. In brief, after centrifugation at 2200 rpm for 20 minutes at 20 ° C using LSM 1077 Lymphocyte Separation Medium (PAA, Pasching, Austria), the intermediate layer consisting of PBMC was washed twice in HANKS's medium (PAA) containing 0.1% BSA and 0.5 mM EDTA. The procedure was repeated twice with DMEM Buffer (PAA) containing 2 mM EDTA and 0.5% BSA. After inhibiting nonspecific antibody binding, the following monoclonal antibodies and appropriate isotype controls were used for flow cytometry: CD3, CD4, CD8, CD56, CD14, CD16, CD56, CD25 and CD19 (all BD). A FACS Canto-II (BD) was used for flow cytometric analysis. The acquired data were analyzed by FlowJo software (TreeStar, Ashland, OR). Numbers of circulating cells were calculated by the percentage of the respective cell subset multiplied by the respective subset of absolute cell count obtained from routine blood count.

### Measurements of cytokines and chemokines

Concentrations of cytokines and chemokines (IL-1β, IL-2, IL-4, IL-5, IL-6, IL-8, IL-10, IL-12p70, IFNγ, G-CSF, TNFα, TNFβ, CCL2, CCL3, CCL4, CXCL9, CXCL10) were measured using Flow Cytomix (eBiosciences) as described before [26]. Fractalkine (CX3CL1) was measured by ELISA (BD) [28].

### Measurements of sIL-2R

Concentrations of sIL-2R were measured using Immulite 1000 IL-2R, a solid-phase, two-site chemiluminescent immunometric assay, according to the manufacturer’s instructions (Siemens, Erlangen, Germany).

### Statistics

Box-and-whiskers plots were used to display data graphically. The box-and-whiskers plots show the statistical summary of the median (bold line), quartiles (boxes), range and extreme values. The whiskers extend from the minimum to the maximum value excluding outside (>1.5 times upper/lower quartile, open circle) and "far out" (>3 time upper/lower quartile, asterixes) values which are displayed separately [29]. Correlations between parameters were assessed by Spearman rank correlation test. Moreover, multivariate regression analyses were performed in order to determine which of the parameters independently influence sIL-2R levels [30]. Comparisons of parameters between two different groups were conducted with the Mann–Whitney-U-test. Comparisons between more than two groups were done with the Kruskal-Wallis analysis of variances (ANOVA), followed by Mann–Whitney-U-tests for post hoc analysis. All analyses were two-tailed and p values <0.05 were considered as statistically significant. The levels of significance are indicated in the figures as followed: * p < 0.05, ** p < 0.01, ***p < 0.001. Statistical analyses were performed using SPSS (SPSS, Chicago, IL).

### Results

#### sIL-2R is elevated in chronic liver disease

**Figure 1:**
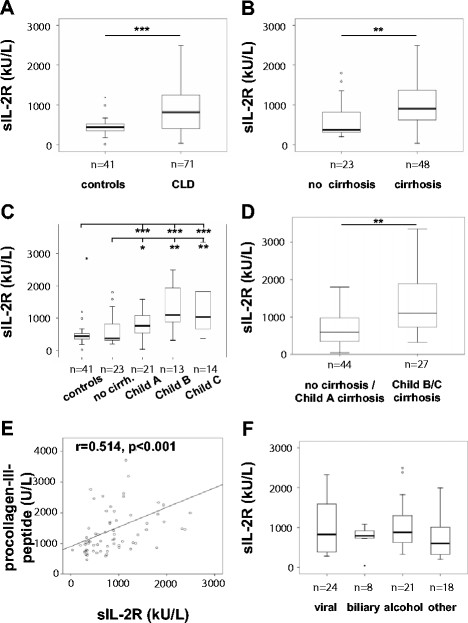
**sIL-2R levels increase in patients with chronic liver disease and are associated with disease progression.** Box plots display serum levels of sIL-2R in kU/L for healthy controls versus patients with chronic liver diseases (**A**), patients without cirrhosis versus cirrhotic patients (**B**), study participants according to Child-Pugh score (**C**), and mild versus advanced CLD (**D**). (**E**) Levels of sIL-2R correlate with procollagen-III-peptide (r = 0.514, p < 0.001, Spearman rank correlation test). (**F**) Serum sIL-2R concentrations do not differ among various etiologies of CLD. Significant differences (Kruskal-Wallis and U-test) are marked by *p < 0.05, **p < 0.01, ***p < 0.001. Open circles and asterixes at the whiskers indicate outlier values.

Serum levels of sIL-2R have been suggested to reflect activation of immune cells in various inflammatory or malignant diseases [1-4,31]. Elevated sIL-2R has been reported for distinct hepatic disorders such as viral hepatitis or primary biliary cirrhosis as well, but the clinical significance of this finding remained vague [23,24]. We therefore measured serum sIL-2R levels in 71 patients with chronic liver diseases (CLD), which had no signs of concomitant infections (clinically, and negative testing for procalcitonin) and no signs of malignancies (especially hepatocellular or cholangiocellular carcinomas). In comparison to 41 healthy controls, CLD patients showed significantly elevated serum sIL-2R levels (median 444 kU/L, range 14–1193, vs. median 818 kU/L, range 39–3976; Figure 1A, Table 1). Importantly, sIL-2R was only increased in patients with established liver cirrhosis (median 374 kU/L, range 204–1795, vs. median 907 kU/L, range 39–3975, Figure 1B), but did not differ between non-cirrhotic patients and controls (Figure 1C). We observed increasing sIL-2R levels with the stage of liver cirrhosis, as assessed by the Child-Pugh score (Figure 1C). In fact, patients with advanced or decompensated cirrhosis, staged Child B and C, had significantly higher sIL-2R concentrations than patients without cirrhosis or with early cirrhosis (median 600 kU/L, range 39–3976, vs. median 1101 kU/L, range 317–3395, Figure 1D). By ROC curve analysis, sIL-2R could discriminate between non-cirrhotic vs cirrhotic patients (area under the curve [AUC] = 0.755, detailed data not shown).

In line, sIL-2R levels correlated inversely with parameters indicating the hepatic biosynthetic capacity, such as albumin (r = −0.496, p < 0.001), pseudocholinesterase activity (r = −0.484, p < 0.001) or international normalized ratio (INR, r = 0.349, p = 0.003, Table 2). Non-invasive markers of liver fibrosis such as hyaluronic acid (r = 0.383, p = 0.003) or procollagen-III-peptide (r = 0.514, p < 0.001, Figure 1E) correlated positively with sIL-2R, emphasizing that sIL-2R is associated with disease progression in CLD patients and may therefore serve as a non-invasive tool to stratify disease severity. Interestingly, serum sIL-2R concentrations were independent of the underlying etiology of chronic liver disease (Figure 1F), indicating that only CLD disease severity, but not the nature of the initial insult determined sIL-2R levels.

**Table 2:** Correlation analysis

	**sIL-2R in liver disease patients**	
	(n = 71)	
	r	*p*
***Liver Function***		
albumin	−0.496	*<0.001*
pseudocholinesterase	−0.484	*<0.001*
international normalized ratio	0.349	*0.003*
bilirubin	0.265	*0.025*
alkaline phosphatase	0.248	*0.037*
***Hepatic fibrosis***		
hyaluronic acid	0.383	*0.003*
procollagen-III-peptide	0.514	*<0.001*
***Inflammation***		
total monocytes	0.366	*0.002*
CD14^++^CD16^-^ monocytes	0.332	*0.006*
CD14^+^CD16^+^ monocytes	0.402	*0.001*
IL-2	0.310	*0.010*
IL-4	0.271	*0.025*
IL-6	0.476	*0.000*
IL-8 (CXCL8)	0.308	*0.011*
IL-10	0.388	*0.001*
IFNγ	0.297	*0.014*
G-CSF	0.239	*0.049*
MIP1α (CCL3)	0.359	*0.002*
MIP1β (CCL4)	0.244	*0.045*
MIG (CXCL9)	0.329	*0.006*
IP-10 (CXCL10)	0.340	*0.005*
fractalkine (CX3CL1)	0.398	*0.001*
***Renal Function***		
cystatin C	0.603	*<0.001*
creatinine	0.301	*0.011*
urea	0.266	*0.026*

sIL-2R serum concentrations were correlated with various routine and experimental laboratory parameters. Only significant correlations are shown. Spearman rank correlation test, p < 0.05.

#### sIL2-R serum levels are associated with circulating monocytes and inflammatory cytokines

**Figure 2:**
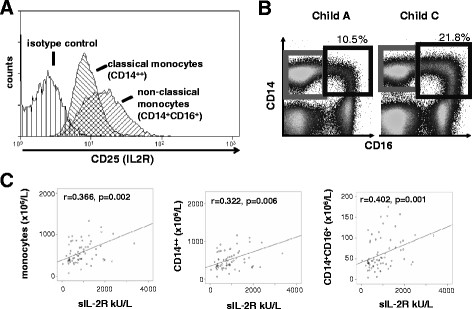
**sIL-2R levels of are associated with circulating monocyte subpopulations.** (**A**) CD25 expression was studied by FACS on circulating monocyte subsets. A representative histogram displays that CD14^+^CD16^+^ monocytes express higher levels of CD25 than the classical CD14^++^ monocytes. (**B**) FACS plots from two representative patients display an increase of CD14^+^CD16^+^ monocytes (right gate) among freshly isolated PBMC. The left plot is derived from a patient with Child A cirrhosis (corresponding sIL-2R 520 kU/L) and the right plot from patient with Child C cirrhosis (sIL-2R 1203 kU/L). (**C**) Serum levels of sIL-2R correlate with numbers of total circulating monocytes (left plot), classical CD14^++^ monocytes (middle plot) and CD14^+^CD16^+^ monocytes (right plot, Spearman rank correlation test).

As a source of circulating sIL-2R, various immune cells including T-cells or monocytes have been postulated [13-17]. We thus analyzed circulating leukocytes in our patient cohort by FACS analysis. Of note, blood monocytes consist of two principal subsets, termed ‘classical’ and ‘non-classical’ monocytes, which are characterized by the expression of CD14^++^ or CD14^+^CD16^+^, respectively [26]. By FACS analysis, we confirmed that not only activated T-cells, but also monocytes expressed IL-2R (CD25) (Figure 2A). In comparison to classical CD14^++^ monocytes, the ‘non-classical’ CD14^+^CD16^+^ monocytes expressed higher levels of CD25 on their surface (Figure 2A). In accordance with previous findings reported by our group [27], monocytes and especially the non-classical (‘proinflammatory’) CD14^+^CD16^+^ monocyte subpopulation increased in patients with liver disease in association with disease progression (Figure 2B, Additional file 1: Figure S1), while NK-, T- and B-lymphocytes were not elevated in CLD patients (Table 1, and data not shown). Serum sIL-2R concentrations did not correlate with NK-, T- or B-lymphocytes or their respective subpopulations, e.g. CD4^+^ or CD8^+^ T-cells. However, sIL-2R levels correlated with circulating monocytes and their subfractions. Serum sIL-2R concentrations correlated not only with total monocyte counts, but also with the classical (CD14^++^CD16^-^) and non-classical (CD14^+^CD16^+^) monocyte subset (Figure 2C, Table 2). The closest correlation was found between sIL-2R and the non-classical CD14^+^CD16^+^ monocytes (r = 0.402, p = 0.001, Figure 2C).

In addition, sIL-2R correlated with various pro-inflammatory cytokines, including IL-2 itself, IFNγ or IL-6, but also with pro-inflammatory chemokines such as CCL3, CCL4, CXCL8, CXCL9, CXCL10, CX3CL1 and others (Table 2). Most of these chemokines have been linked to either monocyte or T-cell activation as well as to trafficking of monocytes or T-cells [32]. However, in our study cohort sIL-2R was more closely correlated to circulating monocytes and especially the ‘inflammatory’ CD14^+^ CD16^+^subset than other cytokines and chemokines (data not shown). Thus, our data indicated that monocytes or macrophages might contribute to circulating sIL-2R levels in patients with chronic liver diseases and that sIL-2R levels are associated with inflammatory processes present in patients with cirrhosis.

#### Renal dysfunction is an additional determinant of sIL2-R serum levels in patients with chronic liver diseases

**Figure 3:**
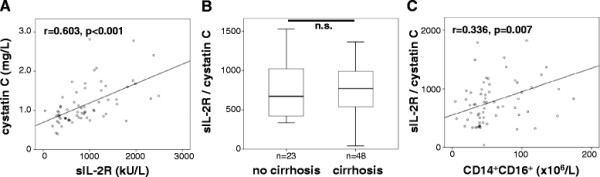
**sIL-2R levels are associated with renal failure.** (**A**) Serum levels of sIL-2R correlate with cystatin C (r = 0.603, p < 0.001, Spearman rank correlation test). (**B**) Box plots display that the ratio of sIL-2R/cystatin C did not differ between CLD patients without or with liver cirrhosis. (**C**) The ratio of sIL-2R/cystatin C correlated with the non-classical CD14^+^CD16^+^ monocytes (Spearman rank correlation test).

Besides its association with inflammatory cells, sIL-2R has been demonstrated to be metabolized and cleared via the kidney in animal models [3]. In line, patients with renal failure were found to have elevated sIL-2R levels as well [33,34]. Due to the fact that decreased glomerular filtration is a condition commonly found in patients with CLD [35], we aimed at verifying the association between sIL-2R levels and liver disease severity as well as circulating monocytes and inflammatory markers by addressing renal function as a confounding factor. Indeed, we found a clear inverse correlation between serum sIL-2R and the glomerular filtration rate as well as positive correlations between sIL-2R and creatinine, cystatin C or urea (Figure 3A, Table 2). When levels of sIL-2R were normalized to renal function by calculating the ‘sIL-2R/cystatin C ratio’, no difference could be detected anymore between patients without or with liver cirrhosis (Figure 3B). However, the correlation between sIL-2R and non-classical monocytes remained significant even after applying the ‘sIL-2R/cystatin C ratio’ (Figure 3C).

In order to assess which of the factors is predominant for regulating sIL-2R levels in patients with chronic liver diseases, a multivariate regression analysis was performed, using sIL-2R as the dependent variable and markers of liver function (albumin, INR, pseudocholinesterase), fibrosis (procollagen-III-peptide), inflammation (IL-6) and renal function (creatinine, cystatin C) and ‘non-classical’ monocytes (CD14^+^CD16^+^) as co-variables. Interestingly, in this multivariate regression analysis, renal dysfunction (cystatin C or creatinine; p < 0.001) and non-classical monocytes (p = 0.029) were the only independently associated variables determining sIL-2R levels in patients with chronic liver diseases (detailed data not shown).

### Discussion

Serum concentrations of the soluble interleukin-2 receptor (sIL-2R) have been studied in many inflammatory or malignant diseases; strikingly, these studies consistently demonstrated that sIL-2R reflects the activation of immune cells [1-4,31]. Only few studies examined sIL-2R in chronic liver diseases, and most of these focused on distinct hepatic disorders such as viral hepatitis or primary biliary cirrhosis [23,24]. Based on the essential pathogenic role of inflammation for the progression of liver injury and fibrosis [32], we hypothesized that sIL-2R might be an indicator of inflammatory cell activation and disease severity in patients with chronic liver diseases (CLD). Strikingly, sIL-2R concentrations were significantly elevated in chronic liver diseases independent of the underlying etiology, associated with the stage of liver cirrhosis and correlated to other established biomarkers of liver function and hepatic fibrosis.

In line with assumptions from non-hepatic inflammatory disorders such as sarcoidosis [2], we speculated that sIL-2R in serum is mainly the result of shedding from the surface of activated immune cells. The thorough comparison of serum sIL-2R levels with flow-cytometric phenotyping of freshly isolated PBMC in CLD patients unexpectedly revealed a close association of sIL-2R with monocytes, specifically with the non-classical CD14^+^CD16^+^ monocyte subset. Several recent studies from animal models have provided evidence that monocytes that infiltrate the injured liver from the circulation are a crucial pathogenic factor for the progression of liver fibrosis [19,36,37]. In order to translate observations from animal models into human pathogenesis, we had previously studied peripheral and intrahepatic monocytes and macrophages from CLD patients and were able to demonstrate that monocytes, especially the non-classical CD14^+^CD16^+^ subset, significantly increase with disease progression [27]. These CD14^+^CD16^+^ monocytes are prone to release important pro-inflammatory cytokines as well as chemokines and are even capable of directly activating collagen-producing hepatic stellate cells, thus suggesting distinct pro-inflammatory and pro-fibrogenic properties of this monocyte subset in vivo [27]. However, the direct flow-cytometric analysis of monocyte subsets is technically demanding, time-consuming and costly [26]. The striking correlation between sIL-2R and (non-classical) monocytes raises the possibility that sIL-2R could serve as a surrogate marker for monocyte subset alterations. It was moreover remarkable that only monocyte and monocyte subpopulations, but not peripheral neutrophils, NK-, B- or T-lymphocytes, correlated with sIL-2R, further indicating that monocytes are an important source of sIL-2R in patients with liver cirrhosis.

However, our study also revealed that sIL-2R levels are strongly influenced by renal function, suggesting a renal clearance of sIL-2R in humans. This is not surprising, because Junghans et al. had shown that the kidney mainly catabolizes sIL-2R in mice, followed by filtration and excretion [3]. We thus used cystatin C as a biomarker to detect early renal impairment in patients with liver dysfunction [35]. In fact, sIL-2R correlated with the glomerular filtration rate calculated from cystatin C, and some of the detected differences for sIL-2R between non-cirrhotic and cirrhotic patients vanished after normalization to renal function. Importantly, the significant correlation between the non-classical monocytes and sIL-2R was unaffected by renal failure, as evidence by normalization calculations as well as multivariate analysis.

### Conclusions

Serum concentrations of sIL-2R are elevated in patients with liver diseases and cirrhosis. Renal failure is an important confounding factor for sIL-2R levels in liver disease patients. sIL-2R levels are associated with cytokine and chemokine concentrations as well as circulating inflammatory cells, especially from the monocyte lineage. These data indicate that sIL-2R might be a potential marker for immune cell activation in CLD, especially for proinflammatory and profibrogenic non-classical CD14^+^CD16^+^ monocytes. Further studies are warranted that evaluate sIL-2R levels prospectively as a potential biomarker for fibrosis progression and that elucidate the possible functional contribution of circulating IL-2 receptor to immune cell attraction or activation.

### Abbreviations

ALT = Alanine aminotransferase activity; CCL = C-C motif chemokine; CLD = Chronic liver disease; CXCL = C-X-C motif chemokine; GFR = Glomerular filtration rate; HBV = Hepatitis B virus; HCV = Hepatitis C virus; IL = Interleukin; INR = International normalized ratio; MELD = Model of end-stage liver disease; n.a. = not applicable / not assessed; NK = Natural killer (cells); PBMC = Peripheral blood mononuclear cells; sIL-2R = soluble interleukin-2 receptor.

### Competing interests

The authors declare no commercial or financial conflict of interest.

### Authors’ contributions

SS, HWZ and FT designed the study, analyzed data and wrote the manuscript, RW performed sIL-2R measurements, CT provided important intellectual content, SS and HWZ collected data, recruited patients and performed FACS analyses. All authors read and approved the final manuscript.

## Pre-publication history

The pre-publication history for this paper can be accessed here:

http://www.biomedcentral.com/1471-230X/12/38/prepub

## Supplementary Material

Additional file 1 **Figure S1.** Frequencies of circulating monocytes and monocyte subsets are elevated in chronic liver disease (CLD). (**A**) The frequencies of total monocytes (defined as CD14^+^ cells) and monocyte subpopulations were measured by FACS analysis. The amount of circulating monocytes relative to all peripheral blood mononuclear cells (PBMC) was significantly higher (***p < 0.001) in patients with CLD than in healthy controls. Single values and median are depicted. (**B**) Absolute numbers of circulating monocytes differ between controls and patients with CLD (*p < 0.05). (**C-D**) Absolute counts of circulating ‘classical’ CD14^++^CD16^-^ (**C**) and ‘non-classical’ CD14 + CD16+ (**D**) monocyte subsets are significantly augmented in patients with CLD (*p < 0.05 and **p < 0.01, respectively). Click here for file
