# Supplementary figures and images for: Elevated circulating soluble interleukin-2 receptor in patients with chronic liver diseases is associated with non-classical monocytes
Source: BMC Gastroenterol. 2012 Apr 24;12:38. doi: 10.1186/1471-230X-12-38 (PMC3434055; doi:10.1186/1471-230X-12-38)

**A****all monocytes**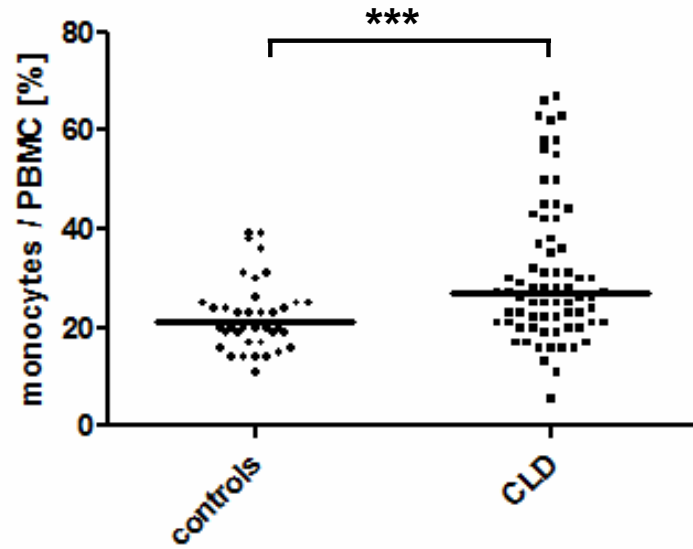**B****all monocytes**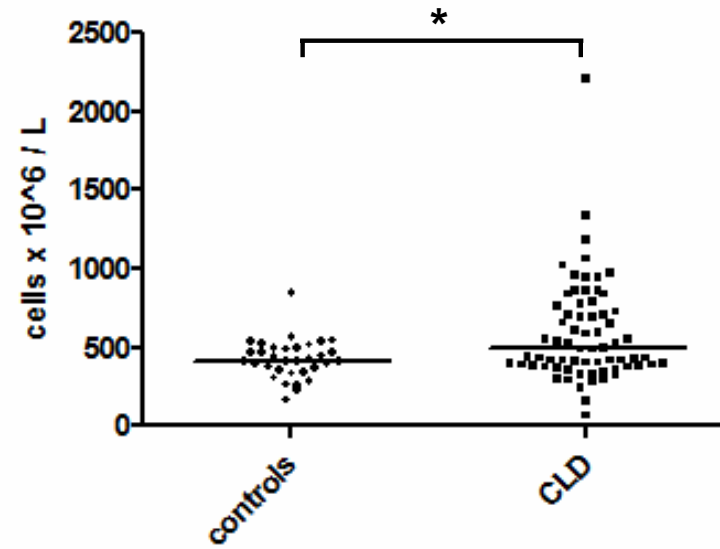**C****CD14<sup>++</sup>CD16<sup>-</sup> monocytes**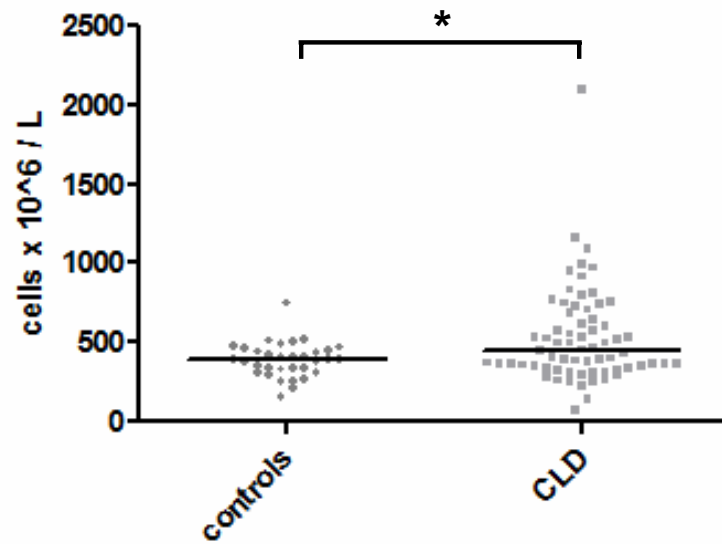**D****CD14<sup>+</sup>CD16<sup>+</sup> monocytes**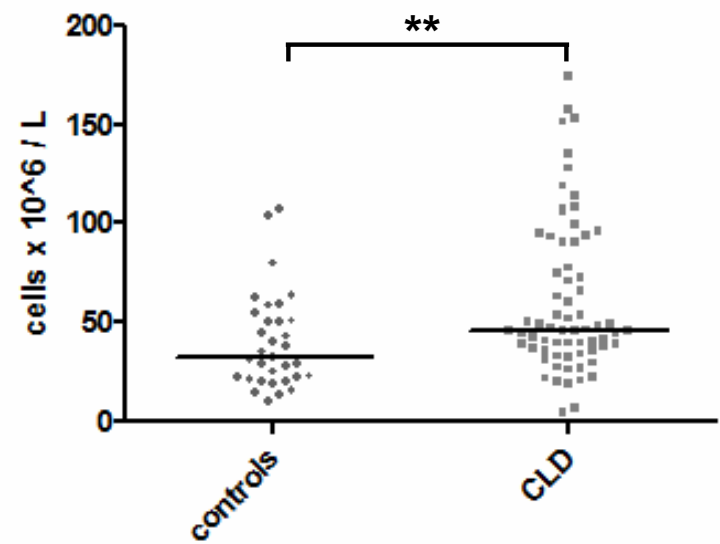

Supplement: Additional file 1 — Figure S1. Frequencies of circulating monocytes and monocyte subsets are elevated in chronic liver disease (CLD). (A) The frequencies of total monocytes (defined as CD14+ cells) and monocyte subpopulations were measured by FACS analysis. The amount of circulating monocytes relative to all peripheral blood mononuclear cells (PBMC) was significantly higher (***p < 0.001) in patients with CLD than in healthy controls. Single values and median are depicted. (B) Absolute numbers of circulating monocytes differ between controls and patients with CLD (*p < 0.05). (C-D) Absolute counts of circulating ‘classical’ CD14++CD16- (C) and ‘non-classical’ CD14 + CD16+ (D) monocyte subsets are significantly augmented in patients with CLD (*p < 0.05 and **p < 0.01, respectively). [file 1471-230X-12-38-S1.pdf]
